# Satisfactory results after endoscopic gluteus medius repair combined with selective gluteus maximus reflected tendon release for the treatment of a full-thickness tear of gluteus medius

**DOI:** 10.1007/s00167-022-07140-x

**Published:** 2022-09-06

**Authors:** Federico Della Rocca, Vincenzo Di Francia, Alberto Giuffrida, Marco Rosolani, Riccardo D’Ambrosi, Alessio D’Addona

**Affiliations:** 1 Humanitas Research Hospital-IRCCS, Via Alessandro Manzoni 36, Rozzano, MI Italy; 2 IRCCS Istituto Ortopedico Galeazzi, Via Galeazzi 4, 20161 Milan, Italy; 3 Dipartimento di Scienze Biomediche per la Salute, Università degli Studi di Milano, Via Mangiagalli 31, Milan, Italy

**Keywords:** Gluteus medius full-thickness tear, Gluteus maximus reflected tendon release, Endoscopy, Hip arthroscopy, Surgical technique

## Abstract

**Purpose:**

The current study aimed to report the mid-term follow-up results of endoscopic gluteus medius repair combined with a systematic release of the gluteus maximus reflected tendon.

**Methods:**

Twenty-two patients with a symptomatic full-thickness tear of the gluteus medius tendon, as diagnosed by clinical examination and imaging (MRI), and who had a failure of conservative treatment for at least 6 months, were retrospectively enrolled for this study. An endoscopic repair of gluteus medius was performed for all patients in combination with gluteus maximus reflected tendon release according to the Polesello technique. The Visual Analogue Scale (VAS) for pain, Modified Harris Hip Score (mHHS), Lower Extremity Functional Scale (LEFS), Hip Outcome Score-Activity Daily Life (HOS-ADL), and Hip Outcome Score-Sport Specific Subscale (HOS-SSS) were administered to each patient before surgery for 6 months, 1 year, and every following year after surgery.

**Results:**

All analysed hip scores (mHHS, LEFS, HOS-ADL, and HOS-SSS) showed statistically significant improvements between the pre-operative and post-operative values at 6 months, 1 year, and the latest follow-up appointments after surgery (*p* < 0.001). The mean pre-operative pain was 8.6 ± 1.0 on the VAS. After surgical treatment, the pain was significantly reduced (*p* < 0.001) on the VAS at 6 months (5.4 ± 1.5), 1 year (4.4 ± 1.8) and the latest follow-up control visit (3.6 ± 2.2). No patient-reported major complications (re-rupture, deep infection or neurovascular injury). Eleven (50%) patients indicated the results as excellent, 7 (32%) as good, 2 (9%) as fair, and 2 (9%) as poor.

**Conclusion:**

The use of abductor tendon repair in combination with a systematic release of the reflected tendon of the gluteus maximus according to the Polesello technique seems to be a safe and effective endoscopic way of treating a full-thickness tear of the gluteus medius.

**Level of evidence:**

Level IV.

**Supplementary Information:**

The online version contains supplementary material available at 10.1007/s00167-022-07140-x.

## Introduction

Recently, greater trochanteric pain syndrome has become a common cause of lateral hip pain. In particular, it is more common amongst women in the 5th and 6th decades of their lives, with a ratio of women to men up to 4:1 [6, 12] and an estimated prevalence of 1.8/1000 [2, 10].

Although trochanteric pain is generally associated with the inflammation of the trochanteric bursa, many studies have suggested that the main cause of lateral hip pain is tearing in gluteal muscles, which is more prevalent in gluteus medius than in gluteus minimus [3, 28]. Abductor tears are characterised more often by the onset of chronic pain generated by attritional forces than by acute traumatic pain [5]. However, partial-thickness undersurface tears may be more common than complete ruptures, and they typically occur at the dual insertion of the anterior and middle muscle fibres of the gluteus medius into the superoposterior and lateral facets of the greater trochanter [11, 12].

Although findings on plain radiographs are generally normal, magnetic resonance imaging (MRI) has been shown to have a specificity of 95% and a sensitivity of 91% in detecting hip abductor tears [19]. Conservative treatment is considered the first option, including steroidal anti-inflammatory drugs, physical therapy, functional therapies, peritrochanteric corticosteroids, and local anaesthetic injections. The surgical option, using either open or endoscopic techniques, is recommended for patients with recalcitrant lateral hip pain who continue to feel pain after more than at least 6 months of non-operative management [12, 23].

The endoscopic procedure was introduced by Voss et al. in 2009, but the literature lacks studies on the gold standard for endoscopic abductor repair techniques [20, 22]. During an endoscopic procedure, the iliotibial band (ITB) overlying the deep gluteal muscles can influence the amount of intra-operative space for viewing, causing peritrochanteric pathology due to frictional forces [22]. Furthermore, the ITB moves over the great trochanter during hip extension, and in the process, the distal border of the gluteal tendons may snap over the great trochanter [24]. Generally, during an endoscopic procedure for gluteus medius repair, the ITB can be split or spared [22]. Polesello et al. performed a release of gluteus maximus reflected tendon to treat symptomatic external snapping hip, creating a space between the ITB and the abductor hip complex [24].

In the current study were reported the outcomes of patients with gluteus medius full-thickness tears repaired endoscopically in combination with a selective release of gluteus maximus reflected tendon according to the Polesello technique [24].

This study aims to demonstrate that endoscopic gluteus medius repair with a selective release of gluteus maximus reflected tendon is a safe, effective, and useful way of improving post-operative outcomes assessed via patient-reported outcomes, satisfaction, and failure rates.

## Materials and methods

The appropriate ethical approval was obtained from the local ethics committee (Humanitas Research Hospital—Protocol Number 618/17).

All procedures involving human participants in this study followed the ethical standards of the institutional and/or the national research committee, as well as the 1964 Helsinki Declaration and its later amendments or comparable ethical standards. The study was conducted following the STROBE checklist for cohort studies [7]. Informed consent to participate in the study was obtained from all the participants.

Between 2015 and 2018, 22 patients were included in this study. The inclusion criterion was a symptomatic full-thickness tear of the gluteus medius tendon diagnosed by clinical examination and imaging (MRI), with a failure of conservative treatment for at least 6 months. The exclusion criteria were an associated injury (chondral or labral defect), bone marrow oedema of the acetabulum, inflammatory patterns (history of arthritis and synovitis), and other previous surgeries on the affected hip.

Clinical examinations and surgical treatments were performed by an experienced hip surgeon in a high-volume, single surgical centre.

The diagnoses and the relative indications for surgical treatment were based on MRI findings and a clinical examination confirmed by the surgeon. All the patients suffered from trochanteric pain and were not responsive to conservative management (for at least 6 months), with evident limping and functional limitations. The clinical examination revealed gait alteration (known as the Trendelenburg sign) with an inconsistent weakness of the gluteus medius and tenderness at the palpation of the anterosuperior area of the great trochanter [3].

A 1.5 Tesla MRI scan, all performed at the same institution (Istituto Clinico Humanitas, Rozzano, Italy), in which a focal discontinuity of the tendon of the gluteus medius with or without proximal part of tendon retraction was observed, was performed on each patient before surgery [15]. In addition, an endoscopic repair of the gluteus medius in combination with the reflected gluteus maximus tendon release was also performed in all patients.

### Surgical technique

The patient is placed supine on a traction table. The limb under the operation is not tractioned but slightly abducted to allow better visualisation of the peritrochanteric space [25]. When repairing a full-thickness gluteus medius (GM) tear, it is generally preferred to use three regular portals, namely the anterolateral (AL) portal, the midanterior (MA) portal, and the distal anterolateral (DAL) portal, and one accessory portal for the positioning of anchors. The DAL portal is placed 3–4 cm distally to the AL portal, whilst the accessory portal is placed more than 2–3 cm posteriorly and 1–2 cm anteriorly to the DAL portal. A well-placed MA portal should lie distal to the gluteus medius muscle belly and proximal to the vastus lateralis, avoiding injury to both structures and facilitating abductor repair.

Fluoroscopy can aid in proper portal placement by confirming the placement directly over the lateral prominence of the greater trochanter. Extra-articular time is performed without the intra-articular diagnostic check, although the portals used are the same as they are for the intra-articular time. After the peritrochanteric compartment is pointed at, the space is distended with 50–70 mmHg of fluid pressure and a 70° scope is introduced through the AL portal into the potential space between the ITB and the greater trochanter. The ITB is split by entering with an arthroscope directly from the portal [14].

At this point, the gluteus maximus tendon insertion on the ITB (reflected tendon) is released according to the Polesello technique [24]. By aiming at just below the vastus ridge under fluoroscopic visualisation, the surgeon avoids iatrogenic damage to the GM insertion. A motorised shaver is then introduced through the DAL portal, and the trochanteric bursa is thoroughly cleared. The bursectomy begins distally at the gluteus maximus insertion directed proximally in a systematic fashion. This allows for easy visualisation of the ITB and the greater trochanter, which defines the lateral and the medial borders of the space. Next, a thorough inspection begins at the gluteus maximus insertion into the linea aspera and vastus lateralis, which should be the distal and posterior extent of any dissection. The sciatic nerve is located 3–4 cm posterior to the gluteus maximus insertion. The gluteus medius muscle and the insertion are then evaluated at the anterior and lateral facets. Both facets and the entire tendon should be inspected and carefully probed. The gluteus minimus is often covered by the gluteus medius and visualising it can be challenging. A switching stick can be used to gently retract the medius muscle to see the tendinous insertion of the gluteus minimus onto the anterior facet. When the tear of the gluteus medius is recognised, it must be evaluated for retraction and reparability by assessing tissue quality and the retraction and mobility of the tendon, similar to the process in the case of tears of the rotator cuff during shoulder arthroscopy. If the tear is eligible for repair, the tendon edge is debrided using a shaver until the healthy tissue is visualised. At this point, the preparation of the bony footprint of the torn tendon is performed; the insertion is cleared of the soft tissue remnants, and the bone is decorticated to the point of bleeding.

Suture anchors are then introduced by the accessory portal. This way, as with the shoulder for a rotator cuff repair, the anchors can be placed in the opposite direction to the tears at a 45° inclination to better achieve a more anatomic orientation of the native footprint, covering the entire bald zone without creating tension. A spinal needle is placed first and positioned with arthroscopic and fluoroscopic guidance to find the ideal location and trajectory of the repair. The repair is then performed using two 5.5 mm Eliquis Anchors (DePuy Mitek, Massachusetts, USA) with two sutures. The anchors are then placed, followed by confirmation with fluoroscopy. The tears of the gluteus medius of the lateral facet are generally repaired with four anchors spaced evenly across the tendon footprint [17]. A penetrator is used to pass the suture through the tendon edge. After two anchors are placed proximally, the horizontal mattress stitches are performed sequentially through the free tendon edge using a suture-passing device with one limb of each suture pulled through the anterior part of the tendon and the other pulled through the posterior part.

Knots are then tied using standard arthroscopic knot tying techniques to anatomically reduce the tendon to the footprint. Then two anchors are placed distally and a side-to-side suture is performed to close the tendons and restore the footprint.

### Post-operative rehabilitation

For all the patients, bearing full weight on the operated limb was not recommended for a month, and a hip cast was used to avoid excessive abduction and extension of the hip for the same duration. A slight abduction and hip flexion of up to 90° were admitted passively after 1 month.

Walking with crutches was admitted with partial weight bearing after 1–3 months post-surgery. Isometric exercises with passive and active mobilisation of the operated limb were admitted from the 3rd month onwards.

Four months after surgery, isotonic exercises and deep hip flexion were allowed. Tapis-roulant and exercise bikes were prescribed to improve the lower limbs’ strength and flexibility.

After 6 months, a return to non-contact sports was allowed [16].

### Data collection

Visual Analogue Scale (VAS) for pain, Modified Harris Hip Score (mHHS), Lower Extremity Functional Scale (LEFS), Hip Outcome Score (HOS)–Activity Daily Life (HOS-ADL), and Hip Outcome Score–Sport Specific Subscale (HOS-SSS) were administered to each patient before surgical treatment at 6 months, 1 year, and every following year after surgery [1, 13, 18, 21].

Patients’ satisfaction after the surgery was recorded in four (poor, fair, good, and excellent) conditions [9].

### Demographics

This study had a 100% patient follow-up with an average of 42 ± 14.5 months (range 24–72 months; median 37 months). Twenty-two patients met the inclusion criteria. The participants included four men and eighteen women with a mean age of 58.6 ± 4.9 years (range 52–69 years). No patient had major complications (re-rupture, deep infection or neurovascular injury). The mean time from the insurgence of the pain to the surgery was 15 ± 7.5 months (range 6–36 months), and the mean BMI (kg/m2) of the examined population was 28.5 ± 3.6 (range 20.6–34.3) (Table 1).

**Table 1:** Characteristics of population

*Patient characteristics*	
Number of patients	22
Mean age	58.6 (range 52–69)
Sex (M/F)	(4/18)
BMI (kg/m^2^)	28.5 (20.6–34.3)
Mean follow-up (months)	42 (range 24–72)
Time from pain insurgence to surgery (mean time)	15 months (range 6–36)
Relapse of symptoms (No of patients)	3/22 (13.6%)
Rate of major complications (re-rupture, deep infection, neurovascular injury, persistent stiffness)	0%
Rate of minor complications (swelling, local paresthesia/anaesthesia, superficial wound infection)	5/22 (22%)
Rate of re-operation	1/22 (4.5%)

In this table demographic elements of the population analysed were recorded. Minor and major complications with the relative rate of relapsed symptoms and re-operation rate were registered

### Statistical analysis

The ANOVA one-way test with Geisser–Greenhouse correction was used to compare the pre-operative and post-operative VASs, mHHS, LEFS, HOS-ADL, and HOS-SSS at 6 months, 1 year, and the latest follow-up after surgery. Significance was set at *p* < 0.05.

A sample of 22 subjects with hip dysplasia was determined to be adequate for the current study, assuming a prevalence of a gluteus medius symptomatic full-thickness tear of 14.5%, a desired total width of 95% confidence interval (CI) of 6.5%, and a type I error of 5% [11]. In addition, sample was increased by five patients to preserve the statistical significance in case of unexpected events. The anticipated prevalence of the gluteus medius pathology and its 95% CI were estimated based on the most recent relevant literature [15].

## Results

### Complications

No patient recorded major complications with the requirement of re-operation. A total of four patients (18.2%) recorded minor complications during follow-up visits. In particular, three patients (13.6%) presented swelling with local hematomas in the surgical area, which healed after 1 month without a delay in recovery of functions. One patient (4.5%) had a little area of dehiscence in one portal, which healed in 3 weeks. No patient-reported stiffness or limitation of range of motion (ROM) of the operated limb. No superficial wound infections were recorded. Five patients (22.7%) experienced a mild delay of recovery due to kinesiophobia without sequelae at the 1 year follow-up control visit.

Three patients (13.6%) had a relapse of symptoms (pain and functional limitation) after surgery. The mean time from surgery to the relapse was 17.6 months (range 1–36 months) without evidence of re-rupture at successive MRI control. Amongst these three patients, one (4.5%) underwent re-operation of total hip arthroplasty after 2 years from the endoscopic repair, even though the surgical treatment had been performed well (Table 1).

### Hip scores

**Fig. 1:**
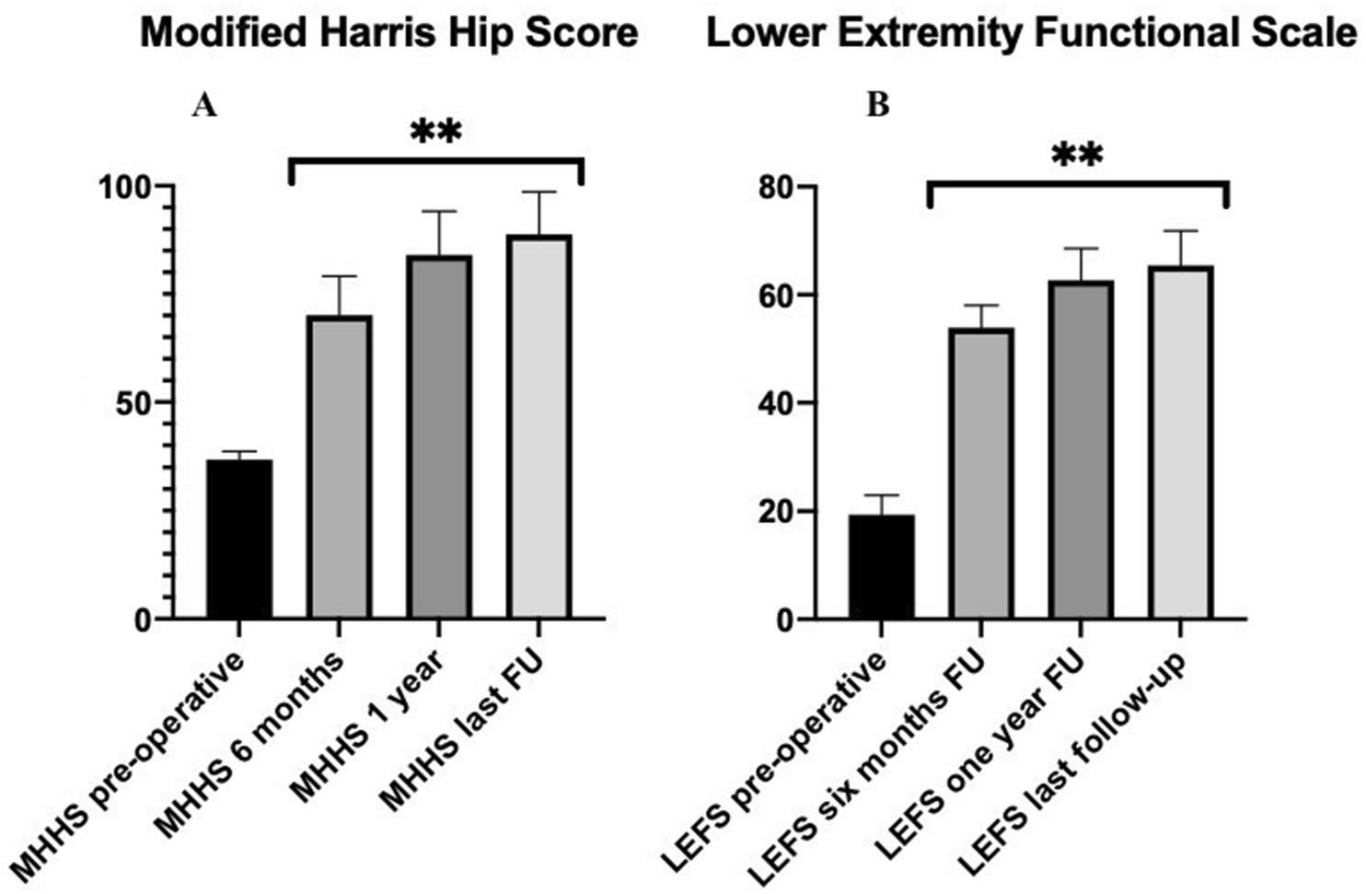
The graph shows **a** the trend of mHHS and **b** LEFS at each follow-up. **Statistical significant improvement compared to the pre-operative value

**Fig. 2:**
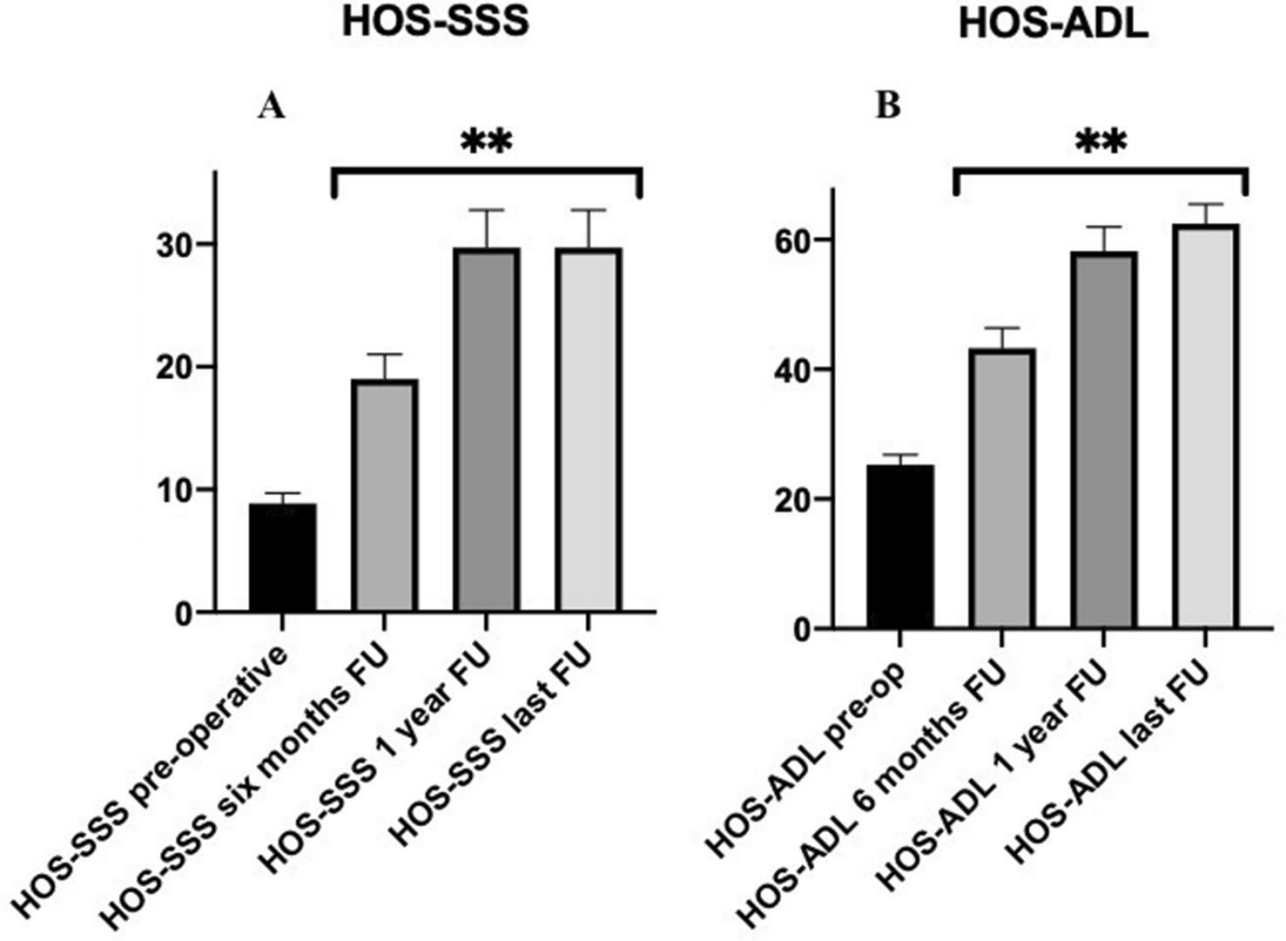
The graph shows **a** the trend of HOS-SSS and **b** HOS-ADL at each follow-up. **Statistical significant improvement compared to the pre-operative value

All analysed hip scores (mHHS [pre-operative: 36.7 ± 1.9; 6 months: 70.1 ± 9.1; 12 months: 84.0 ± 10.1; last follow-up: 88.8 ± 9.8], LEFS [pre-operative: 19.3 ± 3.6; 6 months: 53.1 ± 4.1; 12 months: 62.7 ± 5.8; last follow-up: 65.4 ± 6.3], HOS-ADL [pre-operative: 25.3 ± 1.4; 6 months: 43.3 ± 3.1; 12 months: 58.2 ± 3.8; last follow-up: 62.5 ± 3.0], and HOS-SSS [pre-operative: 8.8 ± 0.8; 6 months: 19.0 ± 2.0; 12 months: 29.7 ± 3.1; last follow-up: 29.7 ± 3.1]) showed statistically significant improvements between the pre-operative and the post-operative values at 6 months, 1 year, and the latest follow-up appointment after surgery (*p* < 0.001) (Figs. 1a, b, 2a, b).

### VAS outcome score and rate of satisfaction

**Fig. 3:**
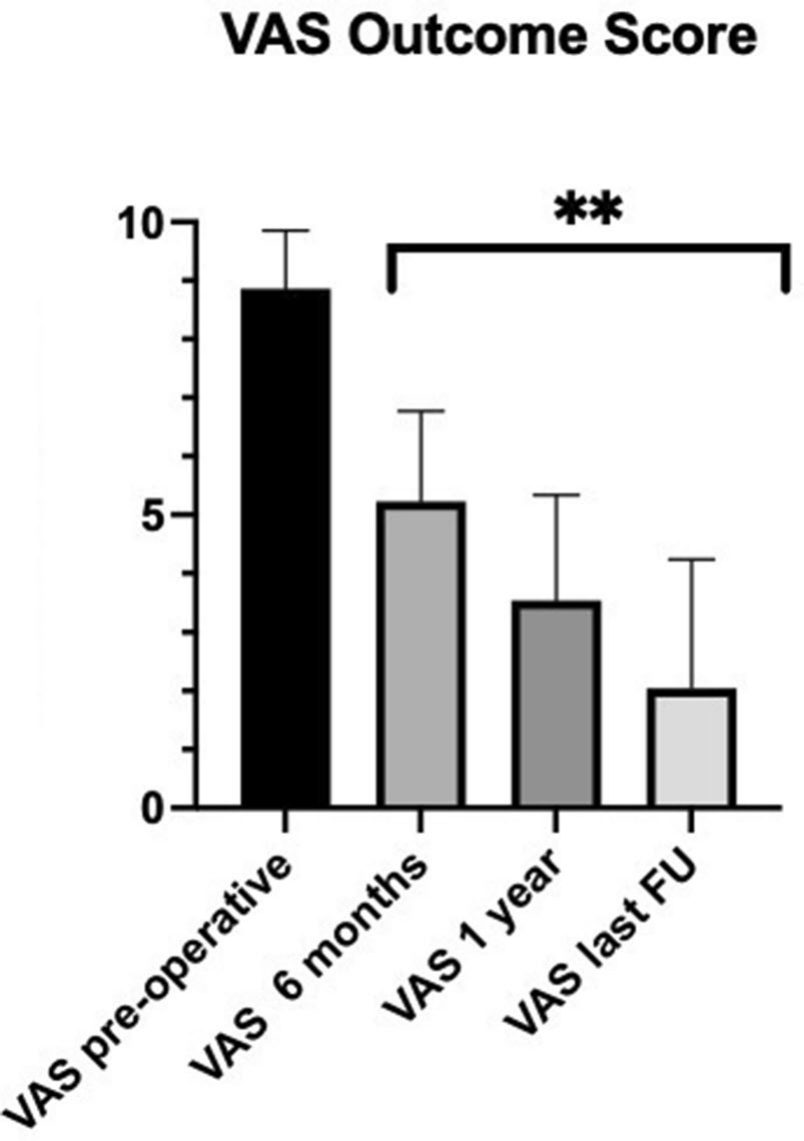
The graph shows the trend of VAS at each follow-up. **Statistical significant improvement compared to the pre-operative value

The mean pre-operative pain was 8.6 ± 1.0 on the VAS. After surgical treatment, the pain was significantly reduced (*p* < 0.001) on the VAS at 6 months (5.4 ± 1.5), 1 year (4.4 ± 1.8) and the latest follow-up control visit after surgery (3.6 ± 2.2) (Fig. 3).

Eleven (50%) patients indicated the results as excellent, 7 (32%) as good, 2 (9%) as fair and 2 (9%) as poor.

## Discussion

The most important finding of the current study confirms that abductor tendon repair in combination with a systematic release of the reflected tendon of gluteus maximus according to the Polesello technique seems to be a safe and effective endoscopic way of treating a full-thickness tear of gluteus medius with promising clinical outcomes.

A full-thickness tear of the gluteus medius tendon is a common cause of pain with limping and functional limitation [28], and surgery is recommended in case of failure of non-operative management [12]. For the first time, the clinical outcomes of this novel surgical procedure combining endoscopic gluteus medius repair with the release of gluteus maximus reflected tendon and medium- and long-term follow-ups have been reported.

First, the primary purpose of the study was to describe this technique. Second, we reported medium- and long-term post-operative outcomes and analysed subjective evaluation scales, rates of satisfaction and re-operation rates.

The rationale of this combined technique is to achieve improvements in detecting symptoms and protect the repaired tendon from frictional forces, excessive compressive loading and stress shielding.

Tendinopathy of the gluteus medius and gluteus minimus tendons is now recognised as a primary local source of lateral hip pain [26], and gluteal tendinopathy is most common in women above 40 years of age. Chronic tendinopathy appears on MRI as increased signal intensity on T2 weighted images [2]. Patients with gluteal tendinopathy may experience pain during prolonged sitting, with subsequent difficulty in standing, particularly if they have been sitting with more than 90° of hip flexion for a long period.

Soslowsky et al. demonstrated in an animal model that compression and high tensile loads combined are more damaging than either stimulus alone. Several factors related to bones and muscles as well as their interactions require consideration for understanding how compressive loading or stress shielding contributes to the underlying pathomechanics of this disorder [27].

The tendons of gluteus medius and gluteus minimus, as well as the associated bursae, can be compressed by the ITB and iliotibial tensing muscles (gluteus maximus, tensor fascia lata and vastus lateralis) at their insertion into the greater trochanter [30].

The excessive hip adductions adopted during static postures and dynamic activities result in an excessive accumulation of compressive tendon loading of hip abductor mechanisms. Higher ranges during hip flexion may also change the ITB tensioning muscles; in fact, the confluence of the ITB with the gluteal fascia into the lumbodorsal fascia contributes to the gluteus medium tendon compression [4, 23, 30].

Considering the pathomechanics of chronic gluteal tendinopathy and the consequent rate of tendinosis and rupture on chronic tendinopathy, it was decided to perform a systematic release of reflected gluteal maximus tendon during GM tendon repair to avoid re-rupture or inflammation of the repaired GM tendon and secondary bursitis from compressive forces of the ITB tensing muscles and post-operative stiffness.

Generally, the Polesello technique is used for pathological external snapping hip onset and consists of an endoscopic gluteus maximus tendon release close to the linea aspera [24].

In their case series, Polesello et al. reported promising excellent results with a high rate of satisfaction (8 patients, 9 hips) and significant improvements in mHHS (*p* = 0.01) from 61.3 preoperatively to 77.6 points at the latest follow-up [24].

It was supposed, with this technique, that the ITB is moved away from the great trochanter to create a larger working space and avoid stiffness post-operatively without limiting the range of motion and secondary snapping hip. Furthermore, moving the hip into abduction increases the space between the ITB and the greater trochanter and facilitates viewing and working in the potential space; this presents a chance for us to better place the anchors without any muscle tensioning forces and with a larger working space.

These findings inform shared decision-making and can help to manage patients’ expectations after surgeries, particularly in patients with a full-thickness gluteus medius tear that is non-responsive to conservative treatment. Furthermore, the results of this study demonstrate how the use of an endoscopic surgical procedure can be considered a valid and effective alternative to non-responsive conservative treatment (physical therapy, injections and rehabilitation). It can help young surgeons who are new to endoscopic and hip arthroscopic surgery.

The main limitations of the current study are the lack of a control group and the relatively small number of patients combined with the retrospective nature of the analysis. Regarding the number of patients, this was in line with the numbers present in the literature, and the number was small mainly because of the relative rarity of this pathology and the strict inclusion/exclusion criteria chosen in this study [15, 24].

A univariate analysis was performed for the study. A recently published paper has demonstrated that multivariate tests do not provide an appreciable increase in power compared to univariate tests [29].

Another limitation is the combination of the two techniques. When two techniques are combined, it becomes difficult to analyse whether the clinical improvement is due to the association of the techniques or one of the two separately. Due to this, further comparative studies on classic endoscopic GM repairs with and without the systematic release of reflected gluteus maximus tendon and with a larger number of participants are required to validate our technique and establish a better surgical recommendation for full-thickness tears of GM.

Finally, another limitations is the use of mHHS, in fact, this scale normally is used for young men with often longstanding severe secondary osteoarthritis after a fracture of the acetabulum, but it is probably the most commonly used outcome measure worldwide.

## Conclusions

Gluteus medius endoscopic repair combined with a systematic release of the reflected tendon of the gluteus maximus according to the Polesello technique is a safe and effective approach to treating a full-thickness tear of the gluteus medius. Gluteus maximus tendon release is useful in creating a larger working space to reach a better positioning of suture anchors. All subjective scales were significantly improved at medium- and long-term follow-up control visits with a high grade of satisfaction by patients, low rate of complications, a return to an active lifestyle, and no rate of re-rupture.

## Supplementary Information

Below is the link to the electronic supplementary material.

Supplementary file1 (PDF 566 KB)
