# Supplementary material for: Satisfactory results after endoscopic gluteus medius repair combined with selective gluteus maximus reflected tendon release for the treatment of a full-thickness tear of gluteus medius
Source: Knee Surg Sports Traumatol Arthrosc. 2022 Sep 6;31(5):2038–45. doi: 10.1007/s00167-022-07140-x (PMC10090025; doi:10.1007/s00167-022-07140-x)
Supplement: Supplementary file 1 — Supplementary file1 (PDF 566 KB) [file 167_2022_7140_MOESM1_ESM.pdf]

HUMANITAS
RESEARCH HOSPITAL

COMITATO ETICO INDIPENDENTE

IRCCS Istituto Clinico Humanitas

Prot. Nr. CE Humanitas ex D.M. 8/2/2013 618/17 18.12.2017

IL COMITATO ETICO INDIPENDENTE

Istituito con Delibera in data 30 Settembre 2016 si è riunito in data 18 Dicembre 2017 alle ore 13:00 nei locali dell'Istituto Clinico Humanitas in via - Manzoni n. 56 in Rozzano per valutare la documentazione, inerente la sperimentazione **Registro RCRA** avente per titolo:

"STUDIO MULTICENTRICO PER LO SVILUPPO DI UN REGISTRO CLINICO E RADIOLOGICO DI SOGGETTI CANDIDATI AD ARTROPROTESI, REVISIONE DI ARTROPROTESI ED ALTRE PROCEDURE ORTOPEDICHE NON PROTESICHE"

Documenti esaminati:

Titolo	Ver	Data Ver	File	Nota
Protocollo	1.0		Progetto registro protesi GAP_V1 01.06.2016.pdf	
Informativa e consenso base	1	01-12-2017	20171201 standard consenso informato final.doc	
Bozza convenzione			20171201 standard contratto final.docx	
Informativa e consenso privacy	1	01-12-2017	20171201 standard informativa privacy final.doc	

**Responsabile dello studio**	**Dr. GUIDO GRAPPIOLO**
**Unità Operativa coinvolta**	**CHIRURGIA DELL'ANCA E PROTESICA**

Rozzano, 18 Dicembre 2017

IRCCS
Istituto Clinico Humanitas
Via Manzoni 56
20089 Rozzano (Milano)
Telefono 02.8224.7216
Fax 02.8224.7208

COMITATO ETICO INDIPENDENTE
IRCCS Istituto Clinico Humanitas

Dopo valutazione il Comitato esprime:

☐ PARERE UNICO ☐ PARERE UNICO con riserva di:

☒ PARERE FAVOREVOLE ☐ PARERE FAVOREVOLE con riserva di:

☐ PARERE SOSPESO ☐ PARERE NON FAVOREVOLE

La sospensione di cui sopra è data in funzione di:

Lo scioglimento della riserva è affidato a:

Rozzano, 18 Dicembre 2017

IRCCS
Istituto Clinico Humanitas
Via Manzoni 56
20089 Rozzano (Milano)
Telefono 02.8224.7216
Fax 02.8224.7208

HUMANITAS
RESEARCH HOSPITAL

COMITATO ETICO INDIPENDENTE
IRCCS Istituto Clinico Humanitas

I componenti che il giorno 18 Dicembre 2017 si sono espressi in merito alla sperimentazione Registro RCRA avente per titolo:

"STUDIO MULTICENTRICO PER LO SVILUPPO DI UN REGISTRO CLINICO E RADIOLOGICO DI SOGGETTI CANDIDATI AD ARTROPROTESI, REVISIONE DI ARTROPROTESI ED ALTRE PROCEDURE ORTOPEDICHE NON PROTESICHE"

Avv. Agostino Migone De Amicis – Presidente	
Prof. Carmelo Carlo Stella – Clinico	
Dott. Franco Pazzucconi – Farmacologo	
Dott. Michele Lagioia – Direttore Sanitario	
Dott. Sergio Pellegrino – Competente in Medicina Generale del Territorio	
Dott. Valter Torri – Biostatistico	
Dott.ssa Maria Bellati – Rappresentante Fondazione Humanitas	
Dott.ssa Maria Fazio – Farmacista	
Dott.ssa Claudia Bacci - Farmacista	
Dott.ssa Barbara Miclini – Rappresentante infermieristico	
Dott. Marco Grimaldi – Esperto clinico del settore	
Dott. Riccardo Caccialanza – Esperto in nutrizione	
Dott. Alberto Podestà - Pediatra	
Prof. Stefano Duga – Esperto di genetica	
Prof. Alessandro Zerbi - Clinico	
Dott.ssa Elena Milani – Ingegnere clinico ed esperta di dispositivi	
Prof. Alberto Mantovani – Direttore Scientifico	
Prof. Carlo Selmi - Clinico	
Prof. Giancarlo Rovati – Bioetico	

Il segretario Dott. Michele Tedeschi

Rozzano, 18 Dicembre 2017

IRCCS
Istituto Clinico Humanitas
Via Manzoni 56
20089 Rozzano (Milano)
Telefono 02.8224.7216
Fax 02.8224.7208
